# Respiratory viruses from hospitalized children with severe pneumonia in the Philippines

**DOI:** 10.1186/1471-2334-12-267

**Published:** 2012-10-23

**Authors:** Akira Suzuki, Socorro Lupisan, Yuki Furuse, Naoko Fuji, Mariko Saito, Raita Tamaki, Hazel Galang, Lydia Sombrero, Melisa Mondoy, Rapunzel Aniceto, Remigio Olveda, Hitoshi Oshitani

**Affiliations:** 1 Department of Virology, Tohoku University Graduate School of Medicine, 2-1 Seiryo-machi, Aoba-ku, Sendai, 980-8575, Japan; 2 Research Institute for Tropical Medicine, Department of Health Compound, FILINVEST Corporate City, Alabang, Muntinlupa City, Philippines; 3 Collaborating Research Center for Emerging and Reemerging Infectious Diseases, Department of Health Compound, FILINVEST Corporate City, Alabang, Muntinlupa City, Philippines

## Abstract

**Background:**

Pneumonia remains a leading cause of child death in developing countries. The viruses in severe pneumonia remain poorly defined.

**Methods:**

The study was conducted at the Eastern Visayas Regional Medical Center in Tacloban City, Philippines from May 2008 to May 2009. Patients aged 8 days to 13 years old who were admitted to the Department of Pediatrics with severe pneumonia were enrolled for the study. Upon admission, polymerase chain reaction was performed using nasopharyngeal swabs and blood cultures to detect respiratory viruses and bacteria, respectively.

**Result:**

Among the 819 patients enrolled, at least one virus was detected in 501 cases (61.2%). In addition, 423 cases were positive for a single virus while bacteria were detected in the blood culture sample of 31 cases. The most commonly detected viruses were human rhinoviruses (n = 189), including types A (n = 103), B (n = 17), and C (n = 69), and respiratory syncytial virus (RSV) (n = 165). Novel viruses such as human metapneumovirus, human coronavirus NL63, human bocavirus, and human polyomaviruses WU and KI were also detected. There were 70 deaths, and one or more viruses were detected in 35 (50%) of these cases. Positivity only for influenza A virus (OR = 4.3, 95% CI = 1.3-14.6) was significantly associated with fatal outcome. From the blood culture, *Burkholderia cepacia* group (n = 9), *Streptococcus pneumoniae* (n = 4), *Staphylococcus aureus* (n = 4), *Haemophilus influenzae* (n = 1), and *Salmonella* C1 (n = 1) were also isolated.

**Conclusion:**

Viruses were commonly detected in children with severe pneumonia in the Philippines. Hence, viral etiologies should be considered while developing better effective strategies to reduce child pneumonia-related deaths in developing countries.

## Background

Infant and under-five mortality rates are commonly considered as important health indicators. In 2000, the United Nations introduced the Millennium Development Goal 4 (MDG4) to reduce under-five mortality by two-thirds between 1990 and 2015. The under-five mortality rate has steadily decreased from 90 per 1000 live births in 1990 to 65 per 1000 live births in 2008 [1]. However, the rate remains high in many developing countries [1], and pneumonia is still the leading cause of childhood mortality [2,3],particularly in the developing countries. It is estimated that approximately 2 million children die from pneumonia annually, and 90% of these deaths occur in developing countries [2-4]. However, viral etiologies may be underestimated in cases of severe pneumonia in the resource-limited countries with limited diagnostic facilities. The role of viral pathogens in severe pneumonia has come into prominence as the role of bacterial infection decreases through early case detection, appropriate antibiotic treatment [5] and introduction of conjugate vaccines.

Historically, the common viruses most closely associated with childhood pneumonia have included influenza virus (Flu), respiratory syncytial virus (RSV), parainfluenza virus (PIV), and adenovirus (AdV). The development of molecular techniques facilitated the detection of novel viruses in patients with respiratory infections. Human metapneumovirus (hMPV) was first documented in 2001 [6]. Since then, a series of human coronaviruses (HCoVs), including NL-63 [7], -NH [8], and -HKU-1 [9], a new genogroup of human rhinoviruses (HRVs) called type-C rhinoviruses [10], human bocavirus (HBoV) [11], and human polyomaviruses WU (WUV) and KI (KIV) [12,13] have been reported. We recently demonstrated the presence of hMPV, HCoV-NL63, HCoV-HKU1, HBoV, WUV, and KIV in patients with influenza-like illness in the Philippines [14], but were unable to assess their clinical significance in children with pneumonia. The etiological roles of these novel viruses remain undefined.

In this prospective study, we investigated the association between presence of a virus and severity of disease in hospitalized children with severe pneumonia to provide an indication of causality. With this aim, we detected viral as well as bacterial pathogens in children hospitalized with severe community-acquired pneumonia and assessed the clinical significance of these pathogens.

## Methods

### Study site

The study was conducted at the Eastern Visayas Regional Medical Center (EVRMC) in Tacloban City, Philippines. Tacloban City is the capital of Leyte Province and the regional center for Region VIII, also known as “Eastern Visayas”. The climate of Leyte Province is classified as tropical monsoon, and it usually rains throughout the year with higher rainfall between November and January. Region VIII consists of two main islands, Leyte and Samar, with a population of approximately 4 million (See Additional file 1a and b: Map of the Philippines and Region VIII). This region is generally economically less developed, and the estimated poverty incidence (the proportion of people in the population whose income is lesser than the cost of basic needs) was 40.7%, compared to the national estimate of 26.9% [15]. The estimated under-five mortality rate in 2008 was 64 per 1000 live births in Region VIII, which is significantly higher than the national average of 28 deaths per 1000 live births in urban areas and 46 deaths per 1000 live births in rural areas of the Philippines [16]. EVRMC has a 250-bed capacity including 50 pediatric beds and is the only tertiary care government hospital in Region VIII. Patients were referred from all over the region, but majority of the patients were from Leyte Province, particularly from Tacloban City.

### Study patients and case definitions

From May 2008 to May 2009, patients aged 8 days to 13 years old who visited the Emergency Room of EVRMC and presented with acute respiratory infection were evaluated according to IMCI guidelines [17]. Patients assessed to have severe pneumonia requiring hospitalization were recruited for participation in the study. The definition of severe pneumonia was based on IMCI guidelines. All cases were screened with the presence of cough or difficulty of breathing as the initial assessment. For patients between 8 days to less than 2 months old, entry criteria included fast breathing (more than 60 breaths/min), chest indrawing, cyanosis, or an inability to drink or suck. For patients between 2 months to less than 5 years old, entry criteria included chest indrawing, cyanosis, or an inability to drink or suck. For patients between 5 to 13 years old, entry criteria included co-morbid illness (including malnutrition), failure to feed, moderate to severe dehydration, signs of respiratory failure (chest indrawing, cyanosis, apnea, or sensorial change), or complications such as plural effusion and pneumothorax. The exclusion criteria included patients less than 7 days old to exclude perinatal infection, patients admitted to any other department of EVRMC for another illness and who had developed symptoms of pneumonia while in hospital, or patients who had been admitted to another hospital within the last 3 days prior to present admission to EVRMC to exclude nosocomial infection.

This was a prospective observational study. The antibiotics provided were based on the guidelines of the Philippine Pediatric Society [18]. As supportive therapy, oxygen was provided using a nasal catheter or oxygen mask. Endotracheal intubation was done if necessary and hooked to a manual ambu bag as there was no mechanical ventilator in the pediatric ICU of EVRMC.

### Patient information

The staff of the Pediatric Department were retrained on the IMCI-Acute Respiratory Infection case management protocol prior to initiation of the study [19]. Trained project pediatricians and nurses collected relevant clinical information on admission including information on antibiotics administered during the current episode. They also recorded signs, symptoms and vital signs of enrolled patients twice a day. Thereafter, patient outcomes were classified using the following five categories on the basis of their condition on discharge: “Discharged improved,” “Discharged against medical advice-Improved,” “Discharged against medical advice-deteriorating,” “Died,” and “Others.” “Others” included those who absconded or were transferred to another hospital. For data analysis, we combined “Discharged against medical advice-deteriorating” and “Died” as the fatal or life-threatening outcome group as we did in previous “Bohol study” in the Philippines [20-22].

### Radiological assessment

Anteroposterior and lateral chest radiographs were taken upon admission. Interpretation was performed by a designated radiologist trained in the standard interpretation of chest radiographs for the diagnosis of childhood pneumonia, as developed by the WHO Pneumonia Vaccine Trial Investigators’ Group [23].

### Detection of viruses

Nasopharyngeal swabs were taken from patients using EX-swab 002 (DENKA SEIKEN, Tokyo, Japan), stirred in 3 ml of viral transport medium (VTM), and stored at 4°C until sample transport. Swabs were shipped to the Research Institute for Tropical Medicine (RITM) for virus detection twice a week using the recommended biological transport containers with temperatures maintained at 4°C. The specimens were subjected to PCR within 10 days after sample collection. Common respiratory viruses were targeted. RNA and DNA present in 100 μl of VTM supernatant were purified using the QIAamp MinElute Virus Spin Kit (Qiagen, Hilden, Germany). To synthesize complementary DNA, 11 μl of the final extract was used as a template with superscript III, random hexamers, and RNase inhibitor (all obtained from Invitrogen, Carlsbad, CA). PCR was performed to detect FluA, FluB, RSVs, hMPV, HCoVs, HBoV, WUV, and KIV as previously described [14]. For PIV-1, -2, and −3, the hemagglutinin-neuraminidase region was amplified [24]. For HRVs, the 5′ noncoding region was amplified using the primer pairs DK001 [25] and DK004 [26]. Negative and positive controls were added in each PCR run. For the purpose of our routine quality control, PCR for all positive samples were double checked in the Department of Virology, Tohoku University Graduate School of Medicine.

Serotypes or genotypes of RSVs, HRVs, and AdVs were determined by sequencing the hypervariable region of the G protein gene for RSVs [27], the VP4-VP2 region of HRVs [28], and the hexon region of AdVs [29]. Sequencing was performed using ABI 3730xl with BigDye version 1.1 (Applied Biosystems, Carlsbad, CA), and sequence results were analyzed by MEGA ver. 4 [30] with reference strains from Genbank.

### Detection of bacteria

Blood culture for bacteria was performed by trained medical technologists in EVRMC as previously described [22]. On admission, 1 ml of venous blood was collected aseptically and inoculated into a bottle containing 20 ml of brain-heart infusion broth with 1% gelatin, 0.125% sodium polyanethol sulfonate, and 0.1% agar. Samples were incubated at 36°C for seven days and subcultures were made on sheep blood, chocolate, and MacConkey agar plates after 18 hours and 3, 5, and 7 days of inoculation. Plates were cultured at 36°C in a candle jar for 2 days. Drug sensitivity testing for all isolates was performed by the disk diffusion method based on Clinical Laboratory Standard Institute (CLSI) guidelines and resistant isolates were tested by the E-test for minimum inhibitory concentration (MIC) determination. All isolates were stored in the skim milk at −20°C and transported to RITM for confirmatory tests. Final identification was done in the Department of Microbiology, RITM. This department has been participating in the College of American Pathologists Proficiency Tests for Microbiology, and it has consistently passed the External Quality Assurance System (EQAS) since 2008. For confirmatory test for *Burkholderia cepacia* group, isolates were sent to Miroku Medical Laboratory in Nagano, Japan, and underwent sequencing for 16S rRNA.

### Statistical analysis

All data was encoded into the FileMaker database (FileMaker CA, USA), and statistical analysis was performed using SPSS 18.0J (SPSS Japan, Tokyo, Japan). Univariate analysis was used to determine the viral pathogens associated with fatal outcomes. The risk of fatal outcome for each detected virus and virus combination was evaluated by estimating the odds ratio (OR) based on categorization in the “Discharged against medical advice-deteriorating” or “Died” category.

### Ethical consideration

The study protocol was approved by the Institutional Review Board of Tohoku University Graduate School of Medicine, RITM, and EVRMC. The parents or guardians gave written informed consent for their children to participate in the study.

## Results

### Patients' profiles

During the 13-month study period, a total of 1242 patients were screened and 1043 met with the inclusion criteria. Of the 1043 patients, 224 refused to participate. A total of 819 cases were subsequently enrolled in the study. Background information and physical findings of the 819 cases are summarized in Table 1. Among the 224 patients whose parents or guardians refused enrolment, we collected data only from 48 patients screened since December 2008. The major reason for the refusal was that parents did not permit to collect blood samples from their children (73.0%[35/48]). The ratio of severe pneumonia (67% [32/48]), very severe pneumonia (23% [11/48]), and others (10% [5/48]) was 67:23:10. This ratio was different from that of the enrolled patients (50:44:6). The median age was 9.0 months, and the age distribution was skewed towards younger age groups (See Additional file 2: Age distribution of children hospitalized with severe pneumonia in Eastern Visayas Regional Medical Center, May 2008 to May 2009).

**Table 1:** Background and Clinical Information of children hospitalized with severe pneumonia, Eastern Visayas Regional Medical Center, May 2008 to May 2009

	**n**	**Percentage**
Demographic information		
Male	446	54.5%
Female	373	45.5%
Age in months*		
Median (Interquartile range)	9(3-20)	
Age groups*		
0~5mo	290	35.5%
6~11mo	177	21.6%
1yo	171	20.9%
2yo	78	9.5%
3~6yo	75	9.2%
7yo or higher	27	3.3%
Initial Diagnosis		
Very Severe Pneumonia	364	44.4%
Severe Pneumonia	410	50.1%
Neonatal Pneumonia and others	45	5.5%
Clinical information		
Symptoms and Signs on admission		
Cough	818	99.9%
Chest indrawing	808	98.7%
Decrease in breath sound*	796	97.3%
Difficulty of breathing	671	81.9%
Wheeze*	418	51.1%
Cyanosis*	62	7.6%
Drowsiness*	35	4.3%
Convulsion	50	6.1%
Chest X-ray		
interstitial infiltrate	111	13.6%
consolidation	93	11.4%
interstitial infiltrate and consolidation	64	7.8%
Outcome		
Discharged	550	67.2%
Discharged against medical advice-Improved	151	18.4%
Discharged against medical advice-Deteriorate	18	2.2%
Died	70	8.5%
Others	30	3.6%
Time interval between Onset and Visit^#^		
Median (Interquartile range)	4(3-7)	(unit=days)
Antibiotic prior to admission	264	32.2%
Total	819	

*Data was missing for one case.

^#^Data was missing for two cases.

Wheezing was observed in half of the patients (n = 418, 51.1%). One hundred seventy five patients (21.4%) had interstitial infiltrates on chest x-ray, and 157 patients (19.2%) had consolidation. The median time interval between the onset of illness and admission was 4 days. Furthermore, 32.2% patients (n = 264) had received antibiotics prior to hospital admission. Since 10.7% (n = 88) of the cases deteriorated during hospital admission, they were classified as “Died” (n = 70, 8.5%) or “Discharged against medical advice-deteriorating” (n = 18, 2.2%).

### Detection of viruses

Among the 819 respiratory samples, 501 samples (61.2%) were positive for at least one virus. These included 423 cases (51.6%); 72 cases (8.8%); and 6 cases (0.7%) who were positive for one, two viruses, and three viruses, respectively (Table 2). The most common respiratory virus detected was HRV (total positive cases, 30.5% [250/819]), including type A (HRV-A) (total, 17.3% [142/819]), type B (HRV-B) (total, 3.1% [25/819]), and type C (HRV-C) (total, 10.1% [83/819]). The second most common virus was RSV (total, 24.1% [198/819]), most commonly subtype A (RSV-A) (97% [195/198]). Different subtypes of AdVs (total, 4.0% [33/819]) were detected: AdV-7 (total, 10; single, 6), AdV-3 (total, 8; single, 5), AdV-6 (total, 6; single 1), AdV-2 (total, 2; single, 1), AdV-4 (total, 2; single, 1), AdV-5 (total, 2; single, 1), Adv-41 (total, 2; single, 0), and Adv-12 (total, 1; single, 0). All cases of FluA (total, 2.2% [18/819]) were seasonal influenza A virus (H1N1). In terms of multiple infection, more than 50% of samples positive for AdVs (54.5% [18/33]), WUV (64.0% [16/25]), HBoV (71.4% [5/7]), FluB (72.7% [8/11]), and KIV (75.0% [3/4]) were positive along with other viruses (Table 2).

**Table 2:** Detection of viral pathogens from children hospitalized with severe pneumonia, Eastern Visayas Regional Medical Center, May 2008 to May 2009

		**FluA**	**FluB**	**RSV-A**	**RSV-B**	**hMPV**	**PIVs**	**HRV-A**	**HRV-B**	**HRV-C**	**HCoV-OC43**	**HCoV-NL63**	**AdVs**	**HBoV**	**WU**	**KI**
FluA		12	0	1	0	0	0	2	0	1	0	0	1	0	0	0
FluB		-	3	0	0	0	0	4	0	3	0	0	0	0	0	0
RSV-A		-	-	160	0	0	0	13	6	3	0	0	2	1	5	1
RSV-B		-	-	-	5	0	0	0	0	0	0	0	0	0	0	0
hMPV		-	-	-	-	17	0	1	0	2	0	0	1	0	0	1
PIVs		-	-	-	-	-	8	1	0	0	0	0	1	0	1	0
HRV-A		-	-	-	-	-	-	103	0	0	0	0	5	2	7	0
HRV-B		-	-	-	-	-	-	-	17	0	0	0	0	1	1	0
HRV-C		-	-	-	-	-	-	-	-	69	0	1	2	1	0	0
HCoV-OC43		-	-	-	-	-	-	-	-	-	1	0	0	0	0	0
HCoV-NL63		-	-	-	-	-	-	-	-	-	-	1	0	0	0	0
AdVs		-	-	-	-	-	-	-	-	-	-	-	15	0	0	1
HBoV		-	-	-	-	-	-	-	-	-	-	-	-	2	0	0
WUV		-	-	-	-	-	-	-	-	-	-	-	-	-	9	0
KIV		-	-	-	-	-	-	-	-	-	-	-	-	-	-	1
Single virus positive	423	12	3	160	5	17	8	103	17	69	1	1	15	2	9	1
Double virus positive	72	5	7	32	0	5	3	35	8	13	0	1	13	5	14	3
Triple viruses positive	6	1	1	1	0	1	1	4	0	1	0	0	5	0	2	1
Total Positive Samples	501	18	11	193	5	23	12	142	25	83	1	2	33	7	25	5

Flu indicates influenzavirus; RSV, respiratory syncytial virus; hMPV, human metapneumovirus; PIV, parainfluenzavirus; HRV, human rhinovirus; HCoV, human coronavirus; AdVs, adenoviruses; HBoV, human bovavirus; WUV, human WU polyomavirus; KVI; human KI polyomavirus.

PIVs-positive cases consisted of 11 cases of PIV-3(single=7) and 1 case of PIV-1(single=1).

Detection of Triple viruses: FluA, HRv-A, and PIV=1 case, FluB, HRV-C, and AdV-5=1 case, hMPV, AdV, and KIV=1case, HRV-A, AdV,and WUV=2 cases, HRV-A, RSV-A, and AdV=1 case.

### Seasonality of the viruses

**Figure 1:**
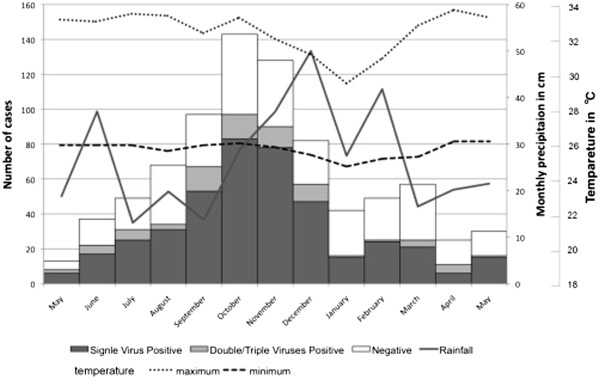
**Monthly Distribution of samples collected from children hospitalized with severe pneumonia, cases positive for viruses, and rainfall precipitation and temperatures (maximum and minimum) in Eastern Visayas Regional Medical Center, May 2008 to May 2009.** The data of the rainfall and temperature was provided by Philippines Atmospheric, Geophysical and Astronomical Service Administration.

**Figure 2:**
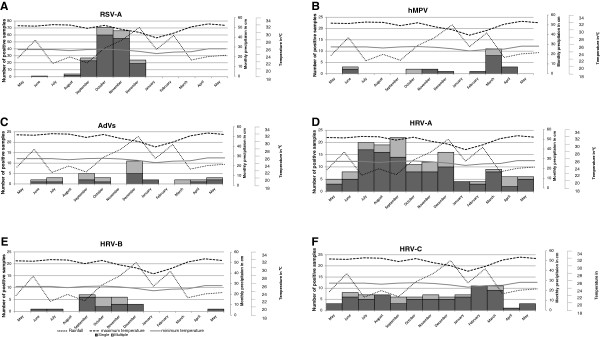
**Monthly distribution of RSV-A(A), hMPV(B), AdVs(C), HRV-A(D), HRV-B(E), and HRV-C(F).** RSV indicates respiratory syncytial virus; hMPV, human metapneumovirus; AdVs, adenoviruses; HRV, human rhinovirus.

The monthly rainfall precipitation and case counts are shown in Figure 1. During the study period, the number of enrolled cases gradually increased and peaked in October, whereas the rainfall increased beginning in October and peaked in December. For viruses detected in more than 20 cases, the monthly distributions of single and multiple virus-positive cases were shown in Figures 2A−2F. RSV-A positivity peaked in October 2009 concurrently with the increase in rainfall in Tacloban City (Figures 1 and 2A). Conversely, there was a cluster of hMPV-positive cases in March which was a month of low rainfall (Figure 2B). Although AdVs were detected intermittently, there was a peak in the number of AdV cases (n = 11) in December (Figure 2C) during which AdV-7 (n = 6), AdV-6 (n = 3), AdV-4 (n = 1), and AdV-12 (n = 1) were detected. There was a cluster of HRV-B-positive cases at the same time as the peak of RSV-A positive cases (Figures 2A and E), whereas HRV-A and HRV-C were detected throughout the study period (Figures 2D and F).

### Associated risk with viral pathogens

Based on previous “Bohol study” in the Philippines, we combined “Discharged against medical advice-deteriorating” and “Died” as the fatal outcome group, as we mentioned earlier, this group denotes the life-threatening cases. Since EVRMC is the only tertiary care government hospital in Region VIII and none of the “Discharged against medical advice-deteriorating” had readmitted, those cases were most likely deceased after discharge. The overall case fatality rate (CFR) was 10.7% [88/819], and one or more viruses were detected in 52.2% [46/88] of these fatal cases (See Additional file 3: Outcome of the children hospitalized with severe pneumonia, Eastern Visayas Regional Medical Center, May 2008 to May 2009). There were fatalities in FluA-, RSV-A-, hMPV-, HRV-, HCoV-, and AdV-positive cases. In terms of CFRs among single virus-positive cases, FluA had the highest CFR (33.3% [4/12]). The CFR of RSV-A was 7.5% [12/160], whereas that of hMPV was 5.9% [1/17]. Among HRVs, HRV-A (14.3% [14/98]) and HRV-B (11.8% [2/17]) had equivalent CFRs but HRV-C had a lesser CFR (5.6% [4/71]). There were two cases positive for HCoVs: a patient with HCoV-OC43 who was classified under the “died” category and one with HCoV-NL63 who was classified under “Discharged against medical advice-deteriorating” category. With respect to AdVs, one AdV-7-positive case was fatal, and two “Discharged against medical advice-deteriorating” patients were positive for AdV-3 and AdV-7. There were two fatal cases each involving two viruses. One case was positive for HRV-A and AdVs, and the other was positive for HRV-B and RSV-A.

The risk of fatal outcome for each detected virus was evaluated on the basis of categorization in the “Discharged against medical advice-deteriorating” or “Died” category. First, cases with at least one virus (8.6% [43/498]) were less likely to be associated with fatal outcomes compared to virus-negative cases (13.9% [42/321]), but there was insufficient evidence of an association between fatal outcome and detection of at least one respiratory virus (OR = 0.62, 95% CI = 0.5-1.0). Second, the risk of fatal outcome according to specific viruses was evaluated (Table 3). Among single virus-positive groups, being positive for FluA virus (odds = 0.50) was associated with a greater risk of fatal outcome (OR= 4.3, 95% CI =1.3-14.6) than non-FluA single virus positive (odds = 0.12).

**Table 3:** **Odds and odds ratio for being fatal outcome**^**#**^**among children hospitalized with severe pneumonia, Eastern Visayas Regional Medical Center, May 2008 to May 2009**

**Odds**^**#**^							
	**Single Virus Positve Cases**		**Multiple Viruses Positve Cases**		**Virus Negative Cases**		**OR**^**$**^**(95 % Cl)**
FluA	4/12	0.50	0/6	0.00	84/801	0.12	4.3(1.3-14.6)
RSV-A	12/160	0.08	1/33	0.03	75/626	0.14	0.6(0.3-1.2)
hMPV	1/17	0.06	0/6	0.00	87/796	0.12	0.5(0.1-3.9)
HRV-A	15/103	0.17	2/39	0.05	71/677	0.12	1.5(0.9-2.7)
HRV-B	2/17	0.13	1/8	0.14	85/794	0.12	1.1(0.3-4.9)
HRV-C	4/69	0.06	0/14	0.00	84/736	0.13	0.5(0.2-1.4)
AdVs	3/15	0.25	2/18	0.13	83/786	0.12	2.1(0.6-7.6)

Flu indicates influenzavirus; RSV, respiratory syncytial virus; hMPV, human metapneumovirus; HRV, human rhinovirus; AdVs, adenoviruses.

^#^Fatal outcome is defined as either in “Discharged against medical advice-Deteriorated” or “Died”.

^$^Single virus positive cases for the virus were compared with all other cases which may include multiple virus positive and virus negative cases.

### Blood culture

Blood culture samples were collected from 814 cases, 31 of which (3.8% [31/814]) were positive in blood culture. Among gram-positive cocci (n = 15), there were *Streptcoccus pneumonia (*n = 4), *Staphylococcus aureus* (n = 4), *Staphylococcus lugdunesis* (n = 3), *Staphylococcus epidermidi*s (n = 2), and *Staphylococcus haemolyticus* (n = 2). Two isolates of *S. aureus* were methicillin resistant *Staphylococcus aureus* (MRSA). For gram-negative rods (n = 3), there was one case for *Haemophilus influenza*, *Enterobacter cloacae*, and *Salmonella* C1. For glucose non-fermentes gram-negative rods (n = 12), there were *Burkholderia cepacia* group (n = 9), *Pseudomonas aeruginosa* (n = 1), *Pseudomonas stutzeri* (n = 1), and *Shewanella putrefaciens* (n = 1). We confirmed isolation of *Burkholderia cepacia* group by 16S rRNA sequencing. For gram-positive rods, there was Bacillus species (n = 1). There were four fatal cases among bacterial culture-positive cases, including *B. cepacia* group (n = 3) and MRSA (n = 1), and one “Discharged against medical advice-deteriorating” case that was positive for MRSA (n = 1). There were concomitant viral infections in samples positive for *S. pneumoniae* (FluB and HRV-A, n = 1), *S. aureus* (HRV-C and RSV-A, n = 1; RSV-A and KIV, n = 1), *MRSA* (HRV-A, n = 2; HRV-A and AdV-2, n = 1), *H. influenzae* (HRV-A, n = 1), and *P. aeruginosa* (RSV-A, n = 1).

## Discussion

Our study highlighted the potential importance of viruses as pathogens of community-acquired pneumonia among hospitalized children in the Philippines. The CFR in our study setting was as high as 8%, which indicated that childhood pneumonia is still an important cause of death in the Philippines. We conducted our current study following the IMCI guideline, because IMCI has been implemented in various developing countries for nearly 20 years [31] as well as we also investigated the causative pathogens in those hospitalized children. Because, the IMCI approach toward childhood pneumonia focuses on bacterial infection, mainly through the empirical use of antibacterial drugs; however, the approach to the management of viral infections is not included in IMCI [19]. Furthermore, IMCI was developed on the basis of etiological studies conducted before 1990, when the detection methods for viral pathogens were suboptimal [32-34]. However, modern molecular methods, such as PCR exhibits higher sensitivity in virus detection. In addition, these methods facilitate the discovery of several new viruses as possible etiological agents for respiratory infection [6-13]. Using these methods, at least one virus was detected in 60.8% of cases in this study. Recent etiological studies also detected viruses in more than 50% of cases in similar settings [35,36].

Although we have demonstrated an important potential role of viral pathogens, economical and effective options for the prevention and treatment of viral infections that are practical to implement in developing countries are unavailable. HRV- and RSV-positive cases accounted for approximately 55% of our study population and had significant effects including those on mortality. However, no effective and safe vaccines or antiviral drugs for these viruses are currently available [37]. The monoclonal RSV antibody for prophylaxis has been used for high-risk infants in developed countries, but this antibody is far too expensive to be used in developing countries [38]. FluA had the highest CFR and was a significant risk factor for fatal outcome, which suggests its potential high mortality impact during larger epidemics. Antiviral drugs such as neuraminidase inhibitors and vaccines for influenza are widely available and used in developed countries; however, they are not affordable in most of the developing countries [39]. To reduce childhood mortality due to viral pneumonia, feasible and inexpensive management strategies, such as triaging hypoxic patients with using a pulse oximeter and administering early treatment with oxygen, should be urgently developed [40].

Although we previously demonstrated the presence of novel viruses in the Philippines [14], our current study provided additional insight on novel viruses. One of the significant findings was the detection of HRV-C. In previous studies, the impact of HRV-C on morbidity and mortality was similar to that of HRV-A and was much greater than that of HRV-B [41-43]. In the present study, HRV-C, the prevalence of which was less than that of HRV-A and greater than that of HRV-B, was detected in 10.1% of the total cases and in three fatal cases. These results suggest that HRV-C could be an important viral pathogen in the cause of severe childhood pneumonia in the Philippines. However, its etiological role is still controversial because this virus has also been detected in healthy individuals [42]. Further studies are necessary to define the role of HRV-C in childhood pneumonia. Another novel virus that shares similar epidemiological and clinical features with RSV in industrialized countries is hMPV [44]. Previous studies in other developing countries indicated that the incidence of hMPV was lower than that of RSV [35,36,45]. However, we observed a fatal case of hMPV which might have an impact in our study setting. A series of HCoVs have been discovered by recent techniques [7-9]. Although none of the HCoV-positive cases exhibited improvement during hospitalization, the number of positive cases was very small. Conversely, there was no fatal case among those positive for newly discovered DNA viruses including HBoV, WUV, and KIV which suggest that their impact in our study is insignificant. However, their severity should be carefully interpreted because of the small number of positive cases for these viruses.

Studies on childhood pneumonia were conducted in the Philippines in the 1980s and 1990s [32,33,46]. In addition to those previous studies, subtyping of viral genetic information revealed group-specific epidemiology. Tupasi et al. demonstrated that RSV was the most common viral cause among children admitted with acute lower respiratory infection in the Philippines [33]. This was consistent with our present study as well as with other recent studies in developing countries [35,36,45,47]. RSV can be classified into two major groups; however, their proportions may change during epidemics [48]. Similarly, we demonstrated that the increase in the number of cases between September and December 2008 was primarily due to RSV-A, although RSV-B was also present in the early stage of the epidemic. We may further need to assess the RSV itself to understand the circulation dynamics of the virus [49]. On the contrary, Capeding et al. demonstrated that AdVs were also important viral pathogens in the Philippines [46]. We observed clustering of AdV-7 in genus B and AdV-6 in genus C, both of which are known to cause respiratory infections of varying severities among children [50]. As each genus of AdVs has different clinical and epidemiological features [51], genetic classification will help us understand the epidemiology of AdVs in tropical climates.

Bacteremia was observed in 3.8% of our cases, and this rate is similar to our previous findings in the Philippines (2.9% and 5%) [20,22]. In addition to two major bacterial pathogens, *S. pneumoniae* (n = 4) and *H. influenzae* (n = 1), the most commonly isolated bacteria was *B. cepacia* group (n = 9). *B. cepacia* is a habitant of the natural environment, but can cause severe infection in the hospital settings especially among immunocompromised patients in ICU [52,53]. Since *B. cepacia* infection in the community is rare [54,55] and the samples were collected upon admission, it is more likely that those isolates of *B. cepacia* group are the contaminants due to contaminated sampling devices, such as disinfectant products [56], rather than causative bacteria pathogen for the pneumonia. However, it should be noted that 3 of those *B. cepacia* group positive-cases died. However, further investigation on the pathogenicity of the *B. cepacia* in our study setting is required.

We might have underestimated the incidence of bacterial pneumonia since prior antibiotic use was observed among 32.4% [264/814] of the enrolled cases. However, a recent study indicated that the prevalence of bacteremia among children with community-acquired pneumonia in the emergency department was as low as 2.1% [57]. Unlike viral isolation, there is no “gold standard” for the diagnosis of bacterial pneumonia among children [58]. Blood culture is widely performed, but its sensitivity is as low as 1-3% [59]. Bacteria cultured from the nasopharynx or throat do not always represent the pathogens in the lungs, as it may include the normal flora of the nose or throat. Lung tap provides an accurate diagnosis of childhood pneumonia [60], but it is an invasive procedure, difficult to perform aseptically, and therefore it is not commonly practiced in developing countries to avoid any iatrogenic complication due to the procedure itself.

The treatment approach of IMCI recommends the empirical use of antibacterial drugs [19], but there is a dilemma of the overuse of antibiotics that increases the emergence of drug resistance. Moreover, antibiotics can be purchased without prescription in the Philippines. For *S. pneumoniae*, all three invasive isolates were susceptible to penicillin, as reported in a previous study in Bohol, Philippines [61]. However, two cases with MRSA did not exhibited improvement during hospitalization. Community-acquired MRSA is a growing problem in developing countries [62]. As IMCI encourages vaccine and antibiotic use for bacterial infections, continuous monitoring of serotype distribution and drug susceptibility is essential for devising a local and cost-effective strategy against childhood pneumonia.

There are some limitations of our study. Firstly, the detection of viruses by molecular techniques may not necessarily indicate that the detected virus is a causative agent of respiratory symptoms since it does not fulfill Koch’s postulate. We understand that the gold standard for the diagnosis of viral infection is virus isolation; however, many novel viruses cannot be isolated, and demonstrating the presence of the viral genome is the only available detection method. On the other hand, we identified multiple viruses in different combinations especially among patients positive for HBoV, WUV, and KIV (Table 2). WUV and KIV are members of *Polyomaviridae* that frequently cause latent infection along with reactivation by immunosupression [63]. Their pathogenicity needs to be elucidated with further epidemiological and clinical studies including appropriate “healthy” control groups. Secondly, we were not able to obtain the demographic information of those who refused to participate in the study due to ethical concerns. The proportion of very severe pneumonia among those who refused to participate was smaller than that of admitted cases, which may have led to an underestimation of the severity of the disease. Thirdly, our study was conducted in the only referral hospital in the region. Therefore, our study population may represent only severe and very severe cases with access to healthcare facilities in the study area. This may have resulted in the underestimation of some etiologies. FluA had the highest CFR, but its prevalence was low. This may be due to the reason that the time interval between onset and admission was 4 days, whereas the viral titer of FluA in respiratory specimens was highest within 48 hours of onset [64].

## Conclusion

Our study demonstrates a potentially important role for viral pathogens in the etiology of severe pneumonia among the children in the Philippines. Our findings suggest that viral etiologies should be always kept in mind while developing effective strategies to reduce child pneumonia-related deaths in developing countries.

## Abbreviations

Flu: Influenza virus; RSV: Respiratory syncytial virus; hMPV: Human metapneumovirus; PIV: Parainfluenzavirus; HRV: Human rhinovirus; HCoV: Human coronavirus; AdVs: Adenoviruses; HBoV: Human bovavirus; WUV: Human WU polyomavirus; KVI: Human KI polyomavirus; IMCI: Integrated Management of Childhood Illness; MIC: Minimum inhibitory concentration; EQAS: External Quality Assurance System.

## Competing interests

The authors declare that they have no competing interests.

## Authors’ contributions

AS, SL, RO, and HO conceived the study and designed it together with FY, NF, HG, LS, and RA. AS, YF, and NF established viral detection methods, and AS, YF, NF, and HG performed the test. LS and MM established and performed detection analysis for bacteria. RT performed statistical analysis. AS drafted the manuscript with assistance of SL and HO. All authors contributed to the final version of the manuscript, read and approved it.

## Pre-publication history

The pre-publication history for this paper can be accessed here:

http://www.biomedcentral.com/1471-2334/12/267/prepub

## Supplementary Material

Additional file 1 **Map of the Philippines and Region VIII.** (a) Darker shaded area indicates Region VIII. (b) Darker shaded area indicates Tacloban city. Click here for file

Additional file 2 Age distribution of children hospitalized with severe pneumonia in Eastern Visayas Regional Medical Center, May 2008 to May 2009. Click here for file

Additional file 3 **Outcome of the children hospitalized with severe pneumonia, Eastern Visayas Regional Medical Center, May 2008 to May 2009.** Flu indicates influenzavirus; RSV, respiratory syncytial virus; hMPV, human metapneumovirus; PIVs, parainfluenzavirus; HRV, human rhinovirus; HCoV, human coronavirus; AdVs, adenoviruses; HBoV, human bovavirus; WUV, human WU polyomavirus; KVI; human KI polyomavirus. Click here for file
