# Supplementary figures and images for: Respiratory viruses from hospitalized children with severe pneumonia in the Philippines
Source: BMC Infect Dis. 2012 Oct 23;12:267. doi: 10.1186/1471-2334-12-267 (PMC3519714; doi:10.1186/1471-2334-12-267)

(a)

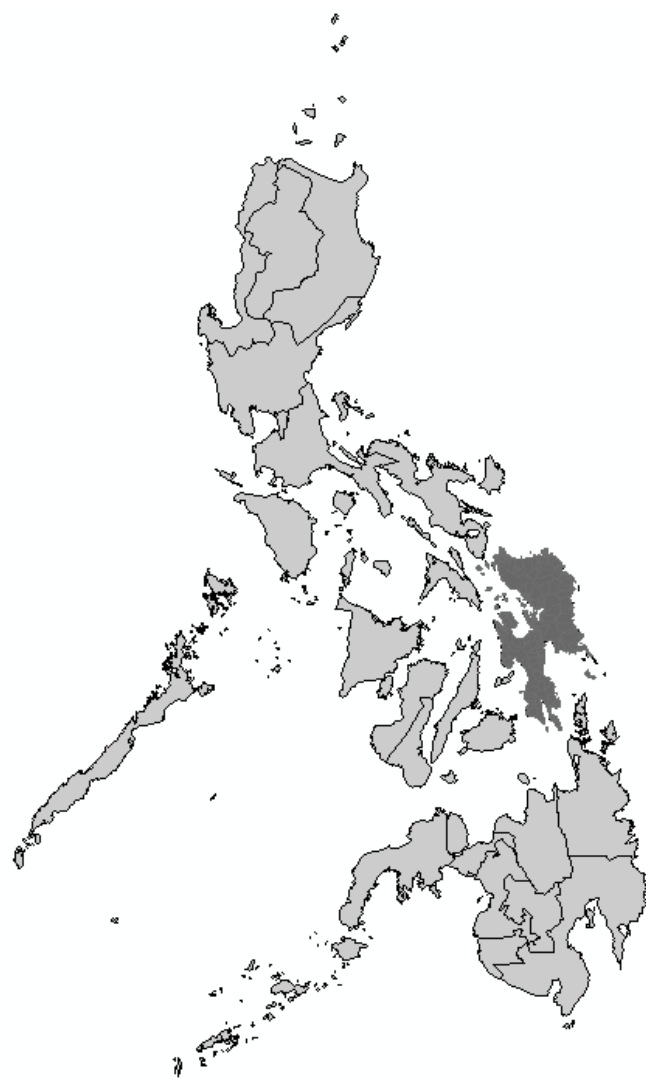

(b)

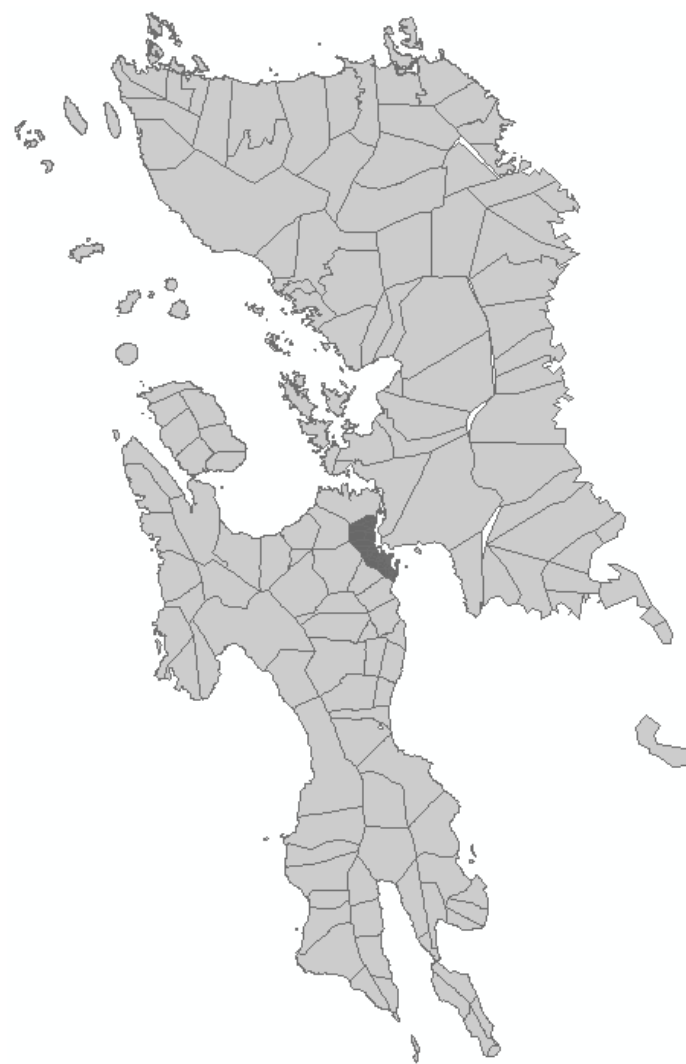

Supplement: Additional file 1 — Map of the Philippines and Region VIII. (a) Darker shaded area indicates Region VIII. (b) Darker shaded area indicates Tacloban city. [file 1471-2334-12-267-S1.pdf]

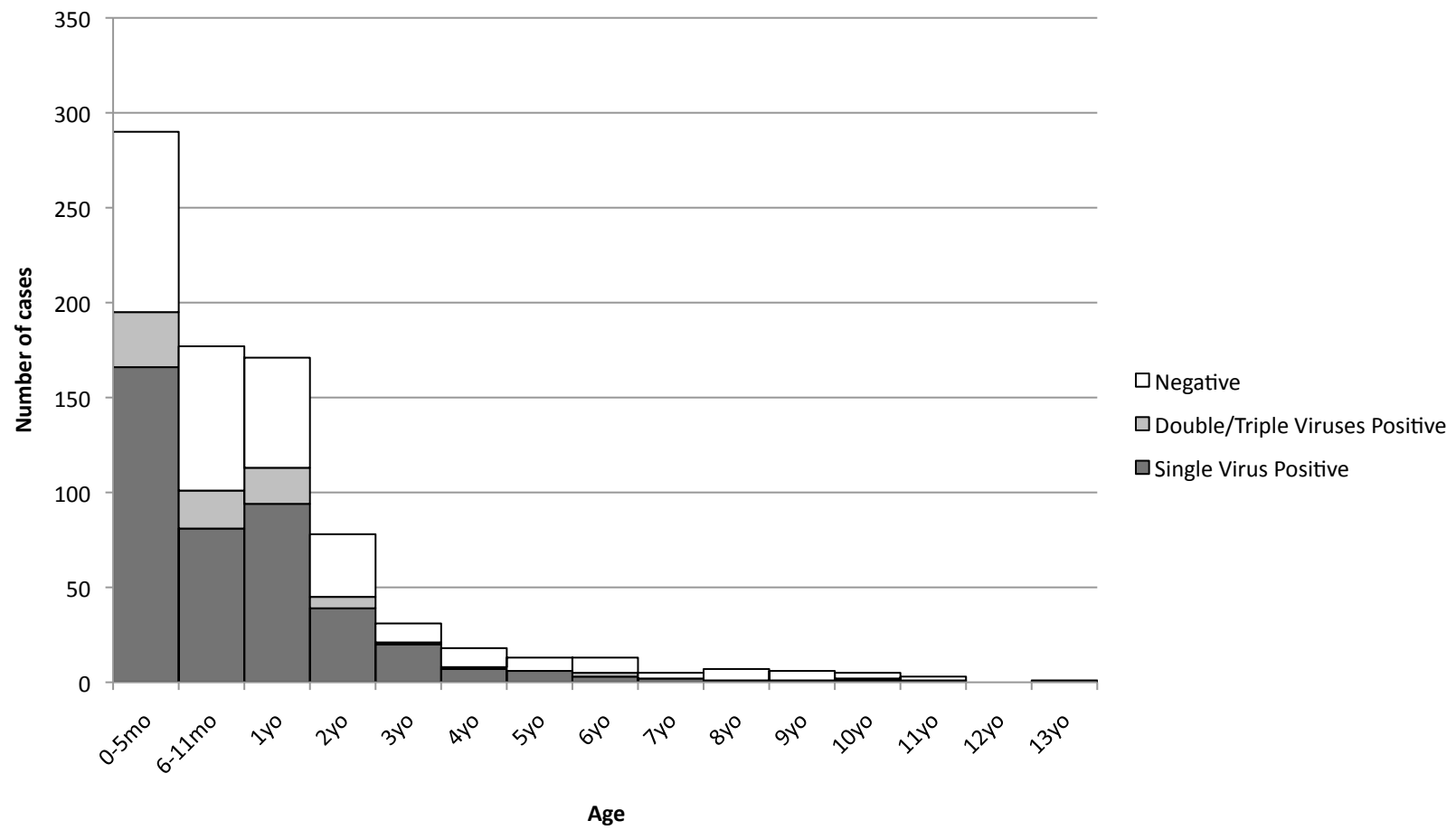

Supplement: Additional file 2 — Age distribution of children hospitalized with severe pneumonia in Eastern Visayas Regional Medical Center, May 2008 to May 2009. [file 1471-2334-12-267-S2.pdf]
