# Supplementary material for: Respiratory viruses from hospitalized children with severe pneumonia in the Philippines
Source: BMC Infect Dis. 2012 Oct 23;12:267. doi: 10.1186/1471-2334-12-267 (PMC3519714; doi:10.1186/1471-2334-12-267)
Supplement: Additional file 3 — Outcome of the children hospitalized with severe pneumonia, Eastern Visayas Regional Medical Center, May 2008 to May 2009. Flu indicates influenzavirus; RSV, respiratory syncytial virus; hMPV, human metapneumovirus; PIVs, parainfluenzavirus; HRV, human rhinovirus; HCoV, human coronavirus; AdVs, adenoviruses; HBoV, human bovavirus; WUV, human WU polyomavirus; KVI; human KI polyomavirus. [file 1471-2334-12-267-S3.pdf]

Additonal file 3. Outcome of the children hospitalized with severe pneumonia, Eastern Visayas Regional Medical Center, May 2008 to May 2009

	Died	Discharged against medical advice-Deteriorated	Discharged against medical advice-Improved	Discharged	Othres	Total
Flu A	3<br>25.0%	1<br>8.3%	2<br>16.7%	6<br>50.0%	0<br>0.0%	12
Flu B	0<br>0.0%	0<br>0.0%	3<br>100.0%	0<br>0.0%	0<br>0.0%	3
RSV-A	10<br>6.3%	2<br>1.3%	28<br>17.5%	117<br>73.1%	3<br>1.9%	160
RSV-B	0<br>0.0%	1<br>20.0%	1<br>20.0%	3<br>60.0%	0<br>0.0%	5
hMPV	1<br>5.9%	0<br>0.0%	3<br>17.6%	12<br>70.6%	1<br>5.9%	17
PIVs	0<br>0.0%	0<br>0.0%	2<br>25.0%	6<br>75.0%	0<br>0.0%	8
HRV-A	12<br>12.2%	2<br>2.0%	13<br>13.3%	67<br>68.4%	4<br>4.1%	98
HRV-B	2<br>11.8%	0<br>0.0%	3<br>17.6%	12<br>70.6%	0<br>0.0%	17
HRV-C	3<br>4.2%	1<br>1.4%	13<br>18.3%	50<br>70.4%	4<br>5.6%	71
HCoV-OC43	1<br>100.0%	0<br>0.0%	0<br>0.0%	0<br>0.0%	0<br>0.0%	1
HCoV-NL63	0<br>0.0%	1<br>100.0%	0<br>0.0%	0<br>0.0%	0<br>0.0%	1
AdVs	1<br>6.7%	2<br>13.3%	4<br>26.7%	8<br>53.3%	0<br>0.0%	15
HBoV	0<br>0.0%	0<br>0.0%	0<br>0.0%	2<br>100.0%	0<br>0.0%	2
WUV	0<br>0.0%	0<br>0.0%	2<br>22.2%	6<br>66.7%	1<br>11.1%	9
KIV	0<br>0.0%	0<br>0.0%	0<br>0.0%	1<br>100.0%	0<br>0.0%	1
Single Virus Positive	33<br>7.9%	10<br>2.4%	74<br>17.6%	290<br>69.0%	13<br>3.1%	420
Double Triple Viruses Positive	2<br>2.6%	1<br>1.3%	19<br>24.4%	53<br>67.9%	3<br>3.8%	78
Non-virus	35<br>10.9%	7<br>2.2%	58<br>18.1%	207<br>64.5%	14<br>4.4%	321
Total Population	70<br>8.5%	18<br>2.2%	151<br>18.4%	550<br>67.2%	29<br>3.5%	819

Flu indicates influenzavirus; RSV, respiratory syncytial virus; hMPV, human metapneumovirus; PIVs, parainfluenzavirus; HRV, human rhinovirus; HCoV, human coronavirus; AdV, adenoviruses; HBoV, human bovavirus; WUV, human WU polyomavirus; KVI; human KI polyomavirus
